# Practical Osmotic Agent for High-Degree Pharmaceutical Pre-Concentration by Organic Solvent Forward Osmosis

**DOI:** 10.3390/membranes14090187

**Published:** 2024-08-29

**Authors:** Ryoichi Takada, Ryosuke Takagi, Zhaohuan Mai, Atsushi Matsuoka, Hideto Matsuyama

**Affiliations:** 1 Asahi Kasei Corporation, Tokyo 100-0006, Japan; takada.rg@om.asahi-kasei.co.jp; 2 Department of Chemical Science and Engineering, Kobe University, Kobe 657-8501, Japan; amatsuoka@people.kobe-u.ac.jp; 3 Research Center for Membrane and Film Technology, Kobe University, Kobe 657-8501, Japan; takagi@harbor.kobe-u.ac.jp (R.T.); zhaohuan.mai@people.kobe-u.ac.jp (Z.M.)

**Keywords:** organic solvent forward osmosis, osmotic agent, high-degree concentration, active pharmaceutical ingredients, polyketone-based thin-film composite hollow fiber membrane

## Abstract

Pre-concentration can reduce the total production costs in the pharmaceutical industry. Organic solvent forward osmosis (OSFO) is a suitable pre-concentration method because of its nonthermal nature, low capital cost, and potential for achieving high-degree concentrations. In a previous study, we first demonstrated a high-degree OSFO concentration. Sucrose octaacetate (SoA) in MeOH was concentrated to 52 wt% using polyethylene glycol (PEG)-400 as the osmotic agent, but the concentrated solution had a concentration of 17% PEG-400 because of the reverse solute flux. This result does not meet the typical purity standards required for pharmaceutical production, indicating the need to determine a suitable osmotic agent that can be used for practical purposes. This study proposes a practical osmotic agent for OSFO pre-concentration. First, osmotic agents were screened from a practical perspective. Polypropylene glycol (PPG)-400 was selected, owing to its low toxicity, good solubility, and low viscosity. Subsequently, the OSFO concentration was demonstrated using PPG-400 as the osmotic agent. SoA in MeOH was concentrated from 9.4 wt% to 48 wt%. The final feed solution contained only 0.04 wt% PPG-400. This result is the first demonstration of successful pharmaceutical pre-concentration using OSFO that satisfies the typical purity requirement in pharmaceutical production.

## 1. Introduction

In the pharmaceutical industry, small-molecule drugs have played a key role in therapeutics for nearly a century and will continue to be the mainstay for the future. The active ingredient of small-molecule drugs, called the active pharmaceutical ingredient (API), is mainly produced in synthetic chemical plants. Multistep chemical reactions are often required to produce an API, with the purification and isolation of intermediates performed for each step, making it extremely cost intensive [1]. Therefore, more economical purification and isolation methods are needed for sustainable drug development.

The addition of a pre-concentration process before purification and isolation reduces the process solution volume to achieve cost-effective purification. Pre-concentration can reduce the capital cost of the subsequent process and shorten the subsequent processing time. Several concentration methods have been used with the prerequisite of maintained quality of the processed liquid. The deterioration of the intermediate is almost not allowed. In addition, the type of impurities that are allowed depends on the nature of the subsequent process. Owing to the above factors, process design with pre-concentration needs to be careful. Nevertheless, the implementation of pre-concentration can drastically reduce the cost of the overall purification process.

Vacuum distillation is the conventional pre-concentration method in API production [2]. This technique reduces the process solution volume to an almost saturated point. Hence, the subsequent process must only handle the minimized process solution. However, most APIs and their intermediates are sensitive to thermal decomposition [2]. Thus, heat treatment should be minimized in API production.

Considering the need for both high-degree concentration and thermal decomposition of the API, organic solvent forward osmosis (OSFO) is a useful method for this situation. OSFO is a membrane-based technology in which the organic solvent permeates owing to the osmotic pressure difference between the draw solution (DS) and feed solution (FS) (the process solution in the case of this study). OSFO is a nonthermal process that can achieve high-degree concentrations when the appropriate DS and membrane are used [3]. Thus, the required capacity of the subsequent process can be minimized by high-degree concentrations, with minimal thermal decomposition of the API. Moreover, unlike reverse osmosis, OSFO does not require the application of external pressure; thus, the initial cost of OSFO would be extremely low. A low-pressure pump, such as a simple liquid-feeding pump, which already exists in a typical pharmaceutical plant, can be used as an OSFO pump. Considering these attributes, the overall cost of the purification process is expected to greatly reduce. However, there are no economic evaluation studies for the social implementation of OSFO as a pre-concentration process. The main reason for the lack of economic evaluation can be ascribed to unsuccessful practical OSFO pre-concentration for the pharmaceutical process.

The main technical challenge in applying OSFO as a pre-concentration process in the pharmaceutical industry is divided into two parts: an organic solvent-resistant membrane and a workable osmotic agent. Various types of organic solvent-resistant membranes have been developed, with some being commercialized. Some commercial membranes can reject small molecules (200–1000 Da) in organic solvents [4]. Therefore, the practical guidelines for developing organic solvent-resistant membranes for OSFO should be determined. However, few research studies have been conducted on osmotic agents that are applicable to OSFO pre-concentration. Cui and Chung first conducted OSFO experiments for pharmaceutical processes using LiCl, methyl palmitate, citric acid, polyethylene glycol 1000 (PEG-1000), and diethanolamine as osmotic agents [5]. Although this pioneering study successfully demonstrated the flux behavior and potential of OSFO in the pharmaceutical industry, the concentration behavior was not observed. Recently, Goh et al. [6] and Li et al. [7] demonstrated the pharmaceutical concentration of OSFO using polyethylene glycol 400 (PEG-400) and an ionic liquid (1-ethyl-3-methylimidazolium bis(trifluoromethylsulfonyl)imide) as osmotic agents, respectively. These studies elucidated the concentration of the API model compound; however, the API concentration was very low (<5 wt%). Practical processes typically employ solutions with concentrations above 5 wt% [8]. Thus, concentrations of 5 wt% or higher are needed to determine the potential of OSFO pre-concentration.

In our previous study [3], a high-degree concentration was first obtained by OSFO using PEG-400 as the osmotic agent. Sucrose octaacetate (SoA) in MeOH solution was successfully concentrated to up to 52 wt%; however, the concentrated solution contained 17 wt% PEG-400 owing to the reverse solute flux (RSF) in OSFO. Such PEG-400 concentrations in the FS are not acceptable for the pre-concentration process in the pharmaceutical industry. To the best of our knowledge, no sufficiently practical osmotic agents for pharmaceutical OSFO pre-concentration have been proposed to date.

In this study, the osmotic agents were screened from the practical aspects as well as the reverse flux point of view. The OSFO performance and RSF of the osmotic agents were evaluated using the selected osmotic agent to discuss the possibility of applying OSFO as a pre-concentration process for practical pharmaceutical processes.

## 2. Screening of Osmotic Agents

MeOH was selected as the representative solvent in pharmaceutical plants for screening osmotic agents because MeOH is a preferred solvent in Pfizer’s solvent selection guide [9] and one of the top three most frequently used solvents in GlaxoSmithKline’s plant [10]. Screening was performed based on the physicochemical properties of the compounds from a practical standpoint.

First, the osmotic agents must be nontoxic because RSF cannot be zero in principle, and the chemicals used in the API production may enter the human body. Cui and Chung [5] suggested that pharmaceutical excipients are nontoxic and promising osmotic agents. In addition to the existing excipients [11,12], other compounds are being studied as candidate excipients [13,14,15]. Among them, neutral compounds with the molecular weight of less than 200 Da were removed because these are difficult to reject through the membrane. Generally, the osmotic pressure is proportional to the molar concentration of the solute. Therefore, DS with a high osmotic pressure is difficult to prepare using high-molecular-weight compounds. Thus, compounds with molecular weights greater than 2000 Da were removed. Based on these points, the selected candidates are listed in Table 1.

Second, high solubility in organic solvents is an essential condition for this application because a higher concentration of osmotic agents can result in a DS with a higher osmotic pressure. Moreover, osmotic agents that are liquids at room temperature (r.t.) are strongly desired, especially when miscible osmotic agents are ideal, because the desired DS concentration can be obtained. As shown in Table 1, PEG-400 and polypropylene glycol (PPG) were selected as candidates. Among them, PEG-400 was removed from the candidates because it leaks from the DS to the FS, as noted in our previous work [3].

A DS with lower viscosity is desirable to decrease the internal concentration polarization (ICP), which decreases the effective osmotic pressure difference between the FS and DS (driving force of OSFO). If the osmotic agent is miscible with water, it can be easily removed from the contaminated API crystals by simple washing with water, even if the API is contaminated with osmotic agents via RSF. Moreover, PPG has a low transaction price (ca. 2 USD/kg) [17]. Considering that even membrane filters are typically single-use in pharmaceutical processes [18,19], the use of DS for disposal would be affordable. Considering these points, PPG-400 was selected as the osmotic agent in this study.

The molecular size of PPG-400 in MeOH was evaluated by molecular dynamics simulations to confirm the possibility of PPG-400 as an osmotic agent with a low RSF. PEG-400 was used as the control osmotic agent. First, PEG-400 and PPG-400 molecules were geometrically optimized using the Forcite module with the condensed-phase optimized molecular potential for atomistic simulation studies II (COMPASS II) force field in Materials Studio 2020 (Figure S1). Subsequently, a system containing each polymer in a MeOH solution was constructed (Figure S2). For each system, 400 MeOH molecules and 10 optimized polymer chains (PEG or PPG, as shown in Figure S1b,d, respectively) were inserted into a cubic box with periodic boundary conditions applied to all dimensions. Geometry optimization was performed at 298 K. Finally, twisted and stretched PEG and PPG molecules were extracted from the MeOH solutions, as shown in Figure S3, and the dimensions of PPG-400 and PEG-400 were determined.

Table 2 lists the dimensions of PEG-400 and PPG-400 in MeOH at 298 K. When a rod- or string-like molecule permeates the small pores, the most important factor affecting the rejection is the major axis of the cross-section of the molecule. A difference of 0.15 Å (0.015 nm) in the size of molecules strongly affects the molecule rejection [20]. The major axis of the cross section of PPG-400 is approximately 0.1 nm larger than that of PEG-400 in both the stretched and twisted chains in MeOH. This suggests a higher rejection of PPG-400 than that of PEG-400, resulting in a lower RSF of PPG-400 than that of PEG-400.

## 3. Experimental Methods

### 3.1. Materials and Chemicals

Porous hollow fibers (HFs), made from polyketones, were supplied by Asahi Kasei Corp. (Tokyo, Japan) as commercially unavailable samples for the fabrication of a thin-film composite (TFC) membrane module, as described previously [3]. For monomers of the interfacial polymerization (IP), 1,3-phenylenediamine (MPD) and 1,3,5-benzenetricarbonyl trichloride (TMC) were obtained from FUJIFILM Wako Pure Chemical Co., Osaka, Japan, and Tokyo Chemical Industry, Tokyo, Japan, respectively. Sodium dodecyl sulfate (SDS) was used as a surfactant for IP, and hexane was used as a solvent; both were obtained from FUJIFILM Wako Pure Chemical Co., Osaka, Japan. SoA, a model API, was obtained from Tokyo Chemical Industry, Tokyo, Japan. MeOH and PPG-400 were obtained from FUJIFILM Wako Pure Chemical Co., Osaka, Japan.

### 3.2. Fabrication of TFC HF Membrane

The membranes were fabricated using the method described in our previous study [3]. An 80-fiber laboratory-scale polyketone HF module with an effective length of 8.0 cm was used as the IP module. An active layer was formed on the bore side via vacuum-assisted IP with MPD, SDS, and TMC. After IP, the TFC-HF membrane module was thermally processed at 50 °C for 5 min and washed at least overnight with deionized (DI) water. After washing, the samples were stored in DI water until use.

### 3.3. Osmotic Pressure Measurement

The osmotic pressure of DS, П (bar), can be calculated from the general expression of osmotic pressure, Equation (1) [21]:

(1) $$П=-\frac{RT}{{\bar{V}}_{MeOH}}ln\left(a_{MeOH}\right)$$

where *R* is the gas constant (0.0831 L bar K^−1^ mol^−1^), *T* (K) is the absolute temperature (296.15 K), ${\bar{V}}_{MeOH}$ (L mol^−1^) is the molar volume of MeOH (0.0737 L mol^−1^ calculated from the molecular weight and the density [22]), and *a_MeOH_* (-) is the activity of MeOH of the sample given by Equation (2):

(2) $$a_{MeOH}=\frac{P_{MeOH,sample}}{P_{MeOH}^{*}}$$

where *P_MeOH,sample_* (kPa) is the MeOH vapor pressure of the sample and P^*^_MeOH_ (kPa) is the vapor pressure of pure MeOH. The vapor pressure of MeOH in the DS was measured using a vapor pressure measurement device established in the laboratory (Figure S4) to determine the activity of MeOH.

### 3.4. OSFO Performance Test

**Figure 1:**
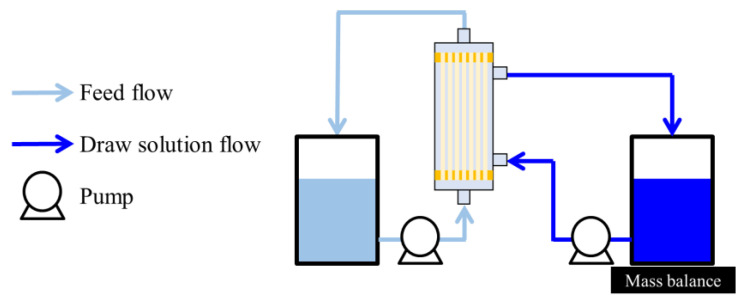
Experimental setup of OSFO.

OSFO performance tests were conducted using the module described in Section 3.2. The experimental setup is schematically illustrated in Figure 1, which shows the FO setup and the so-called active layer (AL)-FS orientation. MeOH solutions of different concentrations (0, 20, 40, and 60 wt%) of SoA were used as the FS. Meanwhile, PPG-400 was used as the osmotic agent. The DS was composed of 2 M osmotic agent and MeOH as the solvent. The tests were conducted at all the SoA concentrations using a single module. Between each test, the FS and DS were replaced. The test lasted for 20 min to minimize changes in the FS and DS composition during the operation. Before the experiment, it was confirmed that the system reached a steady state within a couple of minutes. The initial FS and DS masses were 150 and 1000 g, respectively, with linear velocities of 0.040 and 0.025 m/s, respectively. The test was conducted under ambient temperature (23 ± 3 °C) for at least two modules to assess reproducibility.

The MeOH flux was calculated using Equation (3):

(3) $$J_{MeOH}=\frac{∆m_{d}/\rho_{MeOH}+∆m_{PPGf}/\rho_{PPG}}{A\times ∆t}$$

where *J_MeOH_* (L m^−2^ h^−1^) is the MeOH flux; Δ*m_d_* (kg) is the DS mass variation during the test period Δ*t* (h); Δ*m_PPGf_* (kg) is the PPG-400 mass variation in FS, *A* (m^2^) is the effective area of the module (0.010 m^2^), which is calculated based on the bore surfaces of the fibers; *ρ_MeOH_* (kg L^−1^) is the MeOH density (0.792 kg L^−1^) [22]; and *ρ_PPG_* (kg L^−1^) is the PPG-400 density (1.005 kg/L) [23]. The PPG-400 concentration was determined by liquid chromatography (LC, ACQUITY UPLC I-Class, Nihon Waters Co., Tokyo, Japan)/mass spectrometry (MS, MicrOTOF-QIII, Bruker Daltonics, Billerica, MA, USA). Since the mass variation of DS tank is due to not only the true MeOH permeation but also the mass decrement of PPG in the DS tank (RSF of PPG). The *Dm_PPGf_*/*ρ_PPG_* term corresponds to the RSF of PPG.

### 3.5. High-Degree Concentration by OSFO

A more practical OSFO concentration was demonstrated using PPG-400 as the osmotic agent. The OSFO setup is the same as that described in Section 3.4. SoA was used as the model API with an initial concentration of approximately 9.4 wt%. The test temperature and linear velocity of the FS and DS were the same as those described in Section 3.4. The experiment was conducted for at least two modules to assess reproducibility. The MeOH flux was calculated as described in Section 3.4. The PPG-400 and SoA concentrations were determined using LC (ACQUITY UPLC I-Class, Nihon Waters Co., Tokyo, Japan)/MS (micrOTOF-QIII, Bruker Daltonics, Billerica, MA, USA). The concentration ratio was employed as an indicator of the concentration progress, which was obtained using Equation (4):

(4) $$Concentration\;ratio=\frac{V_{initial}}{V\mathrm{(t)}}$$

where *V(t)* (mL) is the FS volume at the operation time, and *V_initial_* (mL) is the initial FS volume.

## 4. Results and Discussion

### 4.1. Organic Solvent-Resistant Membrane for OSFO

A selective layer was formed on the bore side of the HF support to achieve an effective concentration, as described in a previous study [3]. The characteristics of the HF support are listed in Table S1. The inner diameter of the HF (488 μm) and effective area of the bore surface of 0.010 m^2^ of the module corresponds to a low hold-up volume of 1.2 mL, which is preferable for concentrating a small amount of solution.

**Figure 2:**
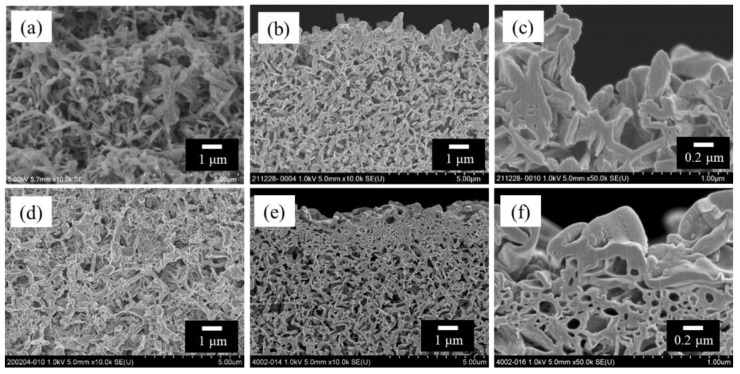
SEM images of polyketone HF support and TFC-HF membrane: (**a**) bore surface of the HF support viewed from the front; (**b**) cross-sectional view near the bore surface of the HF support and (**c**) its high-magnification view; (**d**) bore surface of the TFC-HF membrane viewed from the front; and (**e**) cross-sectional view near the bore surface of the TFC-HF membrane and (**f**) its high-magnification view [3].

Figure 2 shows the scanning electron microscopy (SEM) images of the bore surfaces and cross-sections of the HF support and TFC-HF membrane. Figure 2d–f exhibit the presence of a polyamide-selective layer, which was characterized by attenuated total reflectance–Fourier-transform infrared spectroscopy (Figure S5), indicating the fabricated polyamide layer on the bore surface.

### 4.2. Osmotic Pressure of DS

The osmotic pressure of the DS is an important parameter that determines the FO performance because the osmotic pressure difference between the DS and FS is the driving force of FO. As PPG-400 is a liquid at r.t. and completely miscible with MeOH, as shown in Table 1, PPG-400 of different concentrations in the MeOH solution can be used as a DS. The practical ideal DS concentration would be 100%. With 100% DS, the mixing process of the osmotic agent can be eliminated in pharmaceutical plant operation, and the required DS tank capacity can be minimized. However, for comparison with our previous study that achieved high-degree concentration [3], a PPG-400 concentration of 2 M was used as the DS in this study.

**Figure 3:**
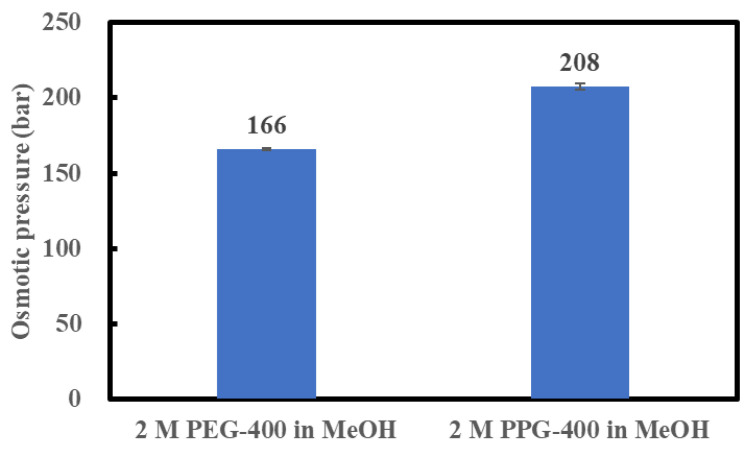
Osmotic pressures of 2 M PEG-400 and 2 M PPG-400 in MeOH.

The osmotic pressures of 2 M PPG-400 in MeOH and 2 M PEG-400 in MeOH were measured at 23 °C, as discussed in Section 3.3, to confirm the possibility of a high API concentration using 2 M PPG-400 in MeOH as the DS. As shown in Figure 3, the osmotic pressure of 2 M PPG-400 in MeOH is higher than that of 2 M PEG-400 in MeOH. As indicated in Equation (1), as the MeOH activity in the DS decreases, the osmotic pressure of the DS increases. In our study, the molar concentration of PEG-400 solution and PPG-400 solution was the same, and molecular weight of the two was also the same. Thus, the higher osmotic pressure of 2 M PPG-400 than that of 2 M PEG-400 can be ascribed to PPG-400’s superior ability to decrease methanol activity in its solution. This confirms the possibility of high-degree concentration using 2 M PPG-400 in MeOH as the DS as 2 M PEG-400 in MeOH succeeded in achieving a high-degree API concentration as the DS [3].

### 4.3. Flux Measurement in OSFO

OSFO performance was measured using PPG-400 as the osmotic agent. The capability of OSFO as a practical pre-concentration method was confirmed using FSs of various SoA concentrations and flux monitoring for 20 min.

**Figure 4:**
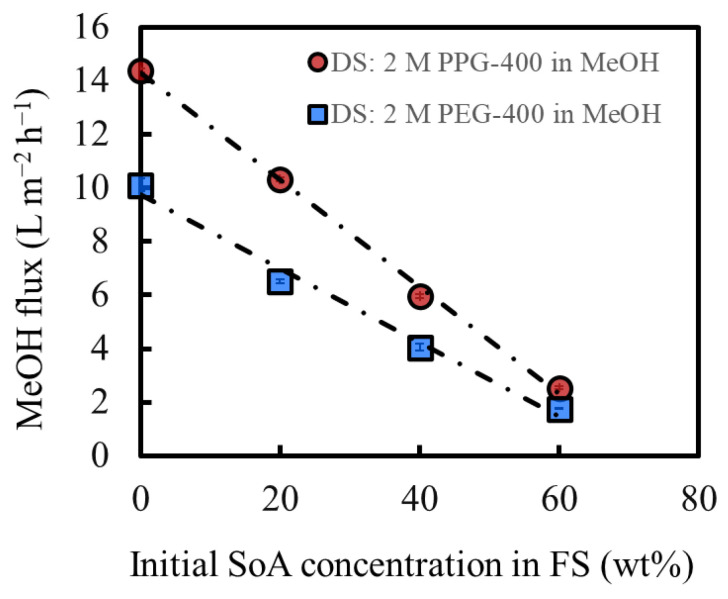
Effect of the initial feed concentration on the solvent flux under OSFO operation; DS: 2 M PPG-400 in MeOH; DS: 2 M PEG-400 in MeOH [3]. (Some error bars are within the symbols).

Figure 4 shows the MeOH flux of OSFO at various SoA concentrations in the FS. For comparison, data from our previous study were used, in which the same experiment was conducted using PEG-400 as the osmotic agent [3]. The MeOH flux decreases as the SoA concentration in the FS increases, which is consistent with the increasing osmotic pressure of the FS with the SoA concentration. The DS of 2 M PPG-400 in MeOH generated a flux even for 60 wt% SoA, confirming that 2 M PPG-400 in MeOH can be used at a high-degree concentration of at least 60 wt%. Moreover, PPG-400 generated a higher flux than PEG-400 at all FS concentrations because of the higher osmotic pressure of 2 M PPG-400 in MeOH than that of 2 M PEG-400 in MeOH (Figure 3) and lower viscosity of PPG-400 than that of PEG-400 (Table 1). It is worth mentioning that the flux exhibits a linear decrease. When there was a pronounced SoA external concentration polarization on the bore surface, the flux would decrease more significantly as the FS concentration increases. Both sets of data suggest the linear velocity of the FS (0.040 m/s) was sufficient for mitigating the SoA external concentration polarization to a practically meaningful extent.

### 4.4. High-Degree Concentration

After confirming the applicability of 2 M PPG-400 in MeOH as a DS at a high-degree concentration, we performed a more practical OSFO with 2 M PPG-400 in MeOH as the DS. The OSFO setup is described in Section 3.5.

**Figure 5:**
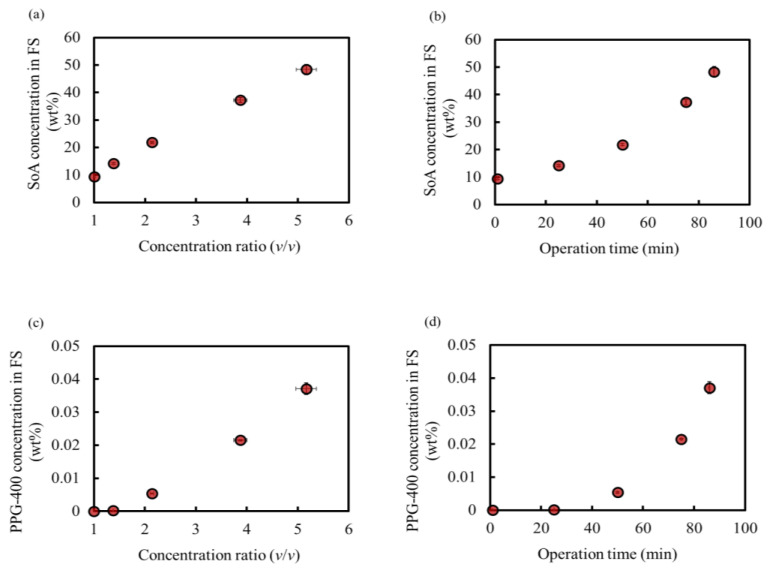
SoA concentration in FS during operation as a function of the (**a**) concentration ratio and (**b**) operation time. PPG-400 concentration in FS during operation as a function of the (**c**) concentration ratio and (**d**) operation time. Initial SoA concentration: 9.4 wt%, DS: 2 M PPG-400 in MeOH. (Some error bars are within the symbols).

Figure 5a,b show the SoA concentration behavior. The SoA concentration in the feed increased proportionally with the concentration ratio, indicating no loss of SoA at this OSFO concentration. Additionally, no SoA was detected in the final DS. Thus, a high concentration of 48 wt% was achieved without any SoA leakage, which is comparable to that in our previous study [3]. This experiment confirmed that PPG-400 could function as a promising osmotic agent for OSFO high-degree concentrations.

Figure 5c,d show the PPG-400 concentration of the FS owing to PPG-400 leakage as a function of the concentration ratio and operation time, respectively.

**Figure 6:**
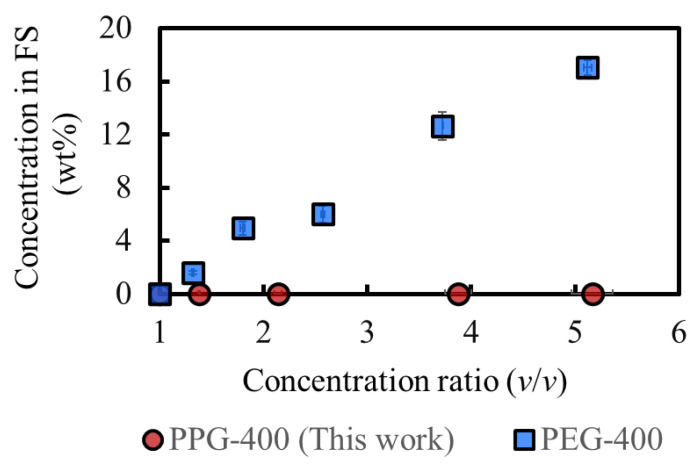
Comparison of the concentrations of osmotic agents in the FS. DS: 2M PPG-400 (this work) and 2M PEG-400 (previous study [3]). (Some error bars are within the symbols).

Figure 6 shows a comparison of the osmotic agents in this study and our previous study, where the same experiment was conducted [3]. The rejection of PPG-400 in MeOH is more prominent than that of PEG-400 in MeOH, as expected from the results of the molecular dynamics simulations (Table 2). The PPG-400 concentration in the final FS is 0.04 wt%, whereas that of PEG-400 is 17 wt%. Assuming the complete removal of MeOH from 100 g FS after processing, 0.04 g PPG-400 and 48 g SoA are expected to remain, and the SoA purity would be over 99.9 wt%. This result satisfies the typical purity requirement (>99 wt%) for the production of APIs and their intermediates [2]. Moreover, additional purification can be conducted by simple washing with water because PPG-400 is completely miscible with water. As a result, PPG-400 was confirmed to have sufficient osmotic pressure to achieve high-degree concentrations and extremely low RSF, with its solubility property promoting the quality of API and its intermediates.

**Figure 7:**
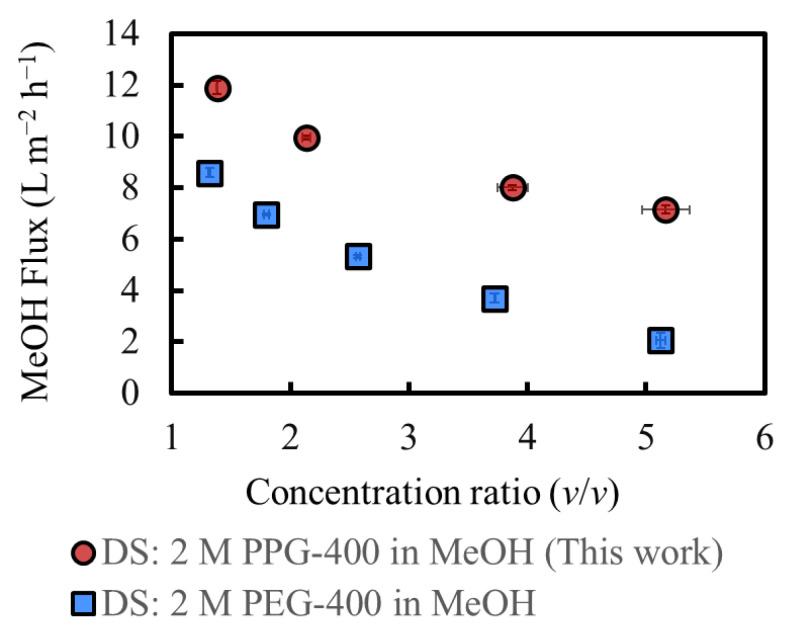
MeOH flux during operation and results of a previous study [3]. Initial SoA concentration: 9.4 wt% (this work) and 11 wt% (previous study). (Some error bars are within the symbols).

Figure 7 shows the MeOH flux during operation, along with the results of a previous study [3]. The flux at the initial stage was approximately 12 L m^−2^ h^−1^ which was consistent with the results presented in Section 4.3. The MeOH flux in this study with PPG-400 as the osmotic agent is higher than that in a previous study with PEG-400 as the osmotic agent [3] at the initial operation stage. Moreover, the flux difference between the two cases increased as the concentration increased, which is consistent with the results of the severe RSF in PEG-400 and almost no RSF in PPG-400, as shown in Figure 6. The increased PEG-400 concentration in the FS increased its osmotic pressure, thereby decreasing the osmotic pressure difference between the DS and FS. In contrast, in this study, the RSF of PPG-400 is negligible. The osmotic pressure of the FS is lower than that of PEG-400, resulting in their higher osmotic pressure difference and higher flux, even for highly concentrated FS. The total amount of MeOH permeated was approximately 120 g. The initial DS amount was 1000 g, and the RSF of PPG-400 was negligible. Thus, the final DS was diluted at a factor of 1.12, which is not considered significant. Such a result is also one of the reasons for the high flux, even for a highly concentrated FS.

The result of this study is the first evidence of the possibility of high-degree pharmaceutical pre-concentration by OSFO that satisfies the typical purity requirement in pharmaceutical production.

## 5. Conclusions

In this study, a practical osmotic agent for pharmaceutical OSFO pre-concentration was explored for the first time. Based on its toxicity, solubility, and viscosity, PPG-400 was selected as a suitable osmotic agent. Further investigation indicated that PPG-400 was expected to perform better than PEG-400 in terms of both RSF and osmotic pressure. Subsequently, OSFO concentration was performed using 2 M PPG-400 in MeOH solution as the DS. As a result, 9.4 wt% SoA in MeOH was successfully concentrated to 48 wt% without SoA leakage. Moreover, the concentrated FS contained only 0.04 wt% PPG-400, corresponding to SoA with a less than 0.1 wt% impurity. This result demonstrated a high-degree pharmaceutical pre-concentration by OSFO that satisfies the typical purity requirement in pharmaceutical production. These results are expected to trigger an economic evaluation of OSFO as a pre-concentration process in the pharmaceutical industry.

## Figures and Tables

**Table 1:** Solubility in MeOH, room temperature phase, viscosity, and solubility in water of the osmotic agents.

Osmotic Agent	Solubility in MeOH at 23 °C (wt%)	Room Temperature Phase	Viscosity (mPa·s) at 30 °C *	Solubility in Water at 23 °C (wt%)
PEG-400	Miscible	Liquid	65	Miscible
NaCl	–	Solid	–	–
KCl	–	Solid	–	–
PEG-600	>90	Solid	–	–
PEG-1000	76–80	Solid	–	–
PEG-2000	57–60	Solid	–	–
PPG-400	Miscible	Liquid	24	Miscible
PPG-700	Miscible	Liquid	69	5
PPG-1000	Miscible	Liquid	101	3
PPG-2000	Miscible	Liquid	225	<1
Polysorbate 20	Soluble [16]	Liquid	ca. 400 [11]	Soluble [16]
Sucrose	–	Solid	–	–
Maltose	–	Solid	–	–

Only the parameters necessary for the screening are presented. * Measured using a viscometer (RE-85L, Toki Sangyo Co., Tokyo, Japan).

**Table 2:** Dimensions of the initial (before dissolution in MeOH) and stretched polymer (PEG and PPG) chains in MeOH solutions.

		Dimension (nm)		
		Length	Width	Height
Initial chain	PEG	3.0	0.4	0.8
	PPG	2.1	0.5	1.0
Stretched chain in MeOH	PEG	2.2	0.6	0.9
	PPG	1.9	0.7	1.0
Twisted chain in MeOH	PEG	1.3	0.9	0.8
	PPG	1.5	0.7	1.0

## Data Availability

The original contributions presented in the study are included in the article and Supplementary Materials, further inquiries can be directed to the corresponding author.

## Supplementary Materials

The following supporting information can be downloaded at: https://www.mdpi.com/article/10.3390/membranes14090187/s1, Figure S1: Molecular models for PEG-400 and PPG-400. (a) Initial model of PEG with molecular weight of 414 g/mol; (b) PEG molecule after geometry optimization; (c) Initial model of PPG with molecular weight of 424 g/mol; (d) PPG molecule after geometry optimization, Figure S2: Molecular models for polymer-methanol solutions at equilibrium. (a) PEG-methanol solution containing 10 PEG chains; (b) PPG-methanol solution containing 10 PPG chains, Figure S3: Dimensional properties of polymers in methanol solutions. L, W, and H represent the length, width and height of the polymer chain, respectively. (a) Twisted PEG molecule in methanol solution; (b) Twisted PPG molecule in methanol solution. (c) Stretched PEG molecule in methanol solution; (d) Stretched PPG molecule in methanol solution, Figure S4: An apparatus for vapor pressure measurement, Figure S5: ATR-FTIR of the bore surface before (HF support) and after the interfacial polymerization (TFC-HF membrane), Table S1: HF support characteristics. References [3,7,24,25,26,27] are cited in Supplementary Materials.
