# Supplementary material for: Practical Osmotic Agent for High-Degree Pharmaceutical Pre-Concentration by Organic Solvent Forward Osmosis
Source: Membranes (Basel). 2024 Aug 29;14(9):187. doi: 10.3390/membranes14090187 (PMC11433671; doi:10.3390/membranes14090187)
Supplement: Supplementary file 1 [file membranes-14-00187-s001.zip › membranes-3127486-supplementary.pdf]

membranes

Supplementary Materials

Practical Osmotic Agent for High-Degree Pharmaceutical Pre-Concentration by Organic Solvent Forward Osmosis

Ryoichi Takada [1,2], Ryosuke Takagi [3], Zhaohuan Mai [3], Atsushi Matsuoka [2] and Hideto Matsuyama [2,3,*]

[1] Asahi Kasei Corporation, 100-0006, Tokyo, Japan
[2] Department of Chemical Science and Engineering, Kobe University, 657-8501 Kobe, Japan
[3] Research Center for Membrane and Film Technology, Kobe University, 657-8501 Kobe, Japan
* Correspondence: matuyama@kobe-u.ac.jp; Tel.: +81-78-803-6180

S1. Molecular Dynamics (MD) Simulations

Both PEG-400 and PPG-400 molecules were modeled by MD simulations using the Forcite module with the Condensed-phase Optimized Molecular Potential for Atomistic Simulation Studies II (COMPASS II) force field in Materials Studio 2020. As shown in Figure S1, each polymer contains two -OH groups at the chain end. The molecular weight of the polymer is 414 g/mol for PEG and 424 g/mol for PPG, respectively.

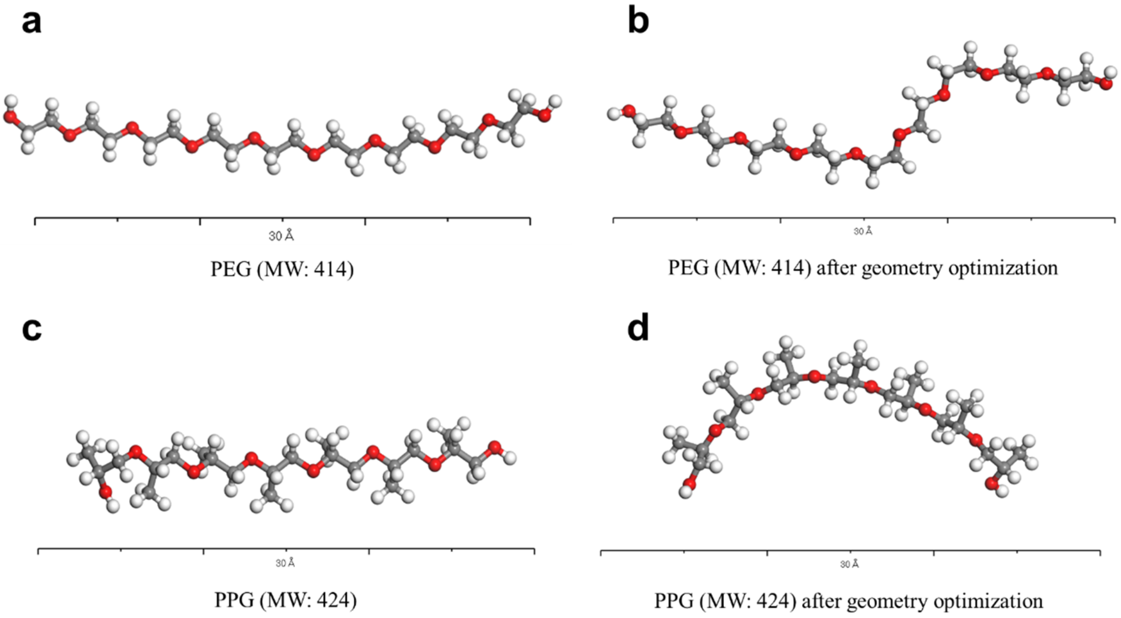

Figure S1. Molecular models for PEG-400 and PPG-400. (**a**) Initial model of PEG with molecular weight of 414 g/mol; (**b**) PEG molecule after geometry optimization; (**c**) Initial model of PPG with molecular weight of 424 g/mol; (**d**) PPG molecule after geometry optimization.

After optimization of PEG and PPG molecules, the system containing each polymer in methanol solution were constructed (Figure S2). For each system, 400 methanol molecules and 10 optimized polymer chains (PEG or PPG as shown in Figure S1b and d, respectively) were inserted into a cubic box with periodic boundary conditions applied in all dimensions. Then a geometry optimization process was first performed, followed by a dynamic run of 500 ps with NVT (constant number of molecules, volume and temperature) ensemble at 298 K. To make sure all systems have reached equilibrium, another run for 500 ps with NPT (constant number of molecules, pressure and temperature) ensemble followed by 5 ns with NVT ensemble were applied, and the energy as well as temperature of both systems reached the steady values. Finally, to compare the structural dimensions

of the two polymers, twisted and stretched PEG and PPG molecules were extracted from methanol solutions as shown in Figure S3.

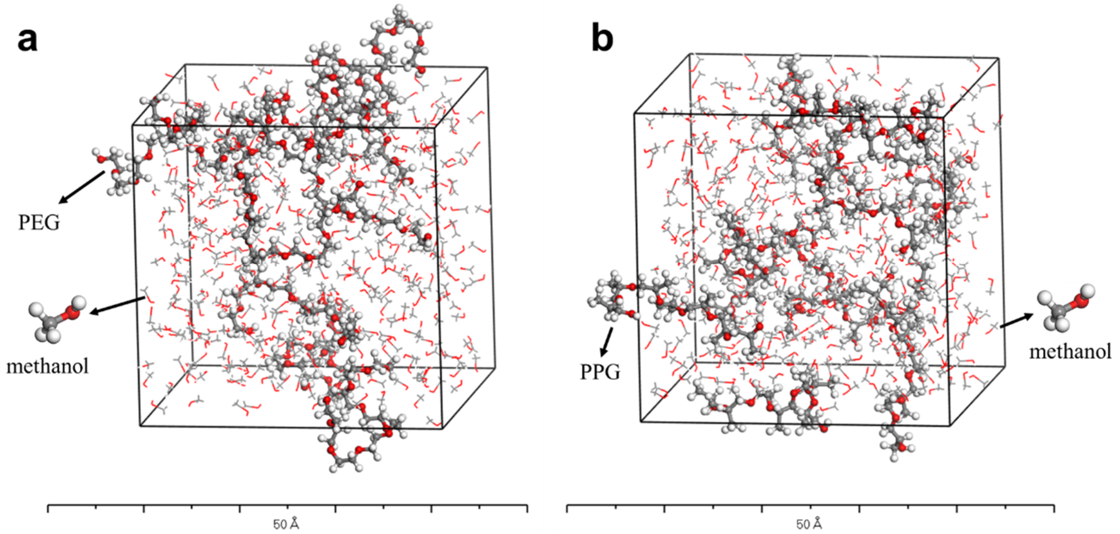

Figure S2. Molecular models for polymer-methanol solutions at equilibrium. (**a**) PEG-methanol solution containing 10 PEG chains; (**b**) PPG-methanol solution containing 10 PPG chains.

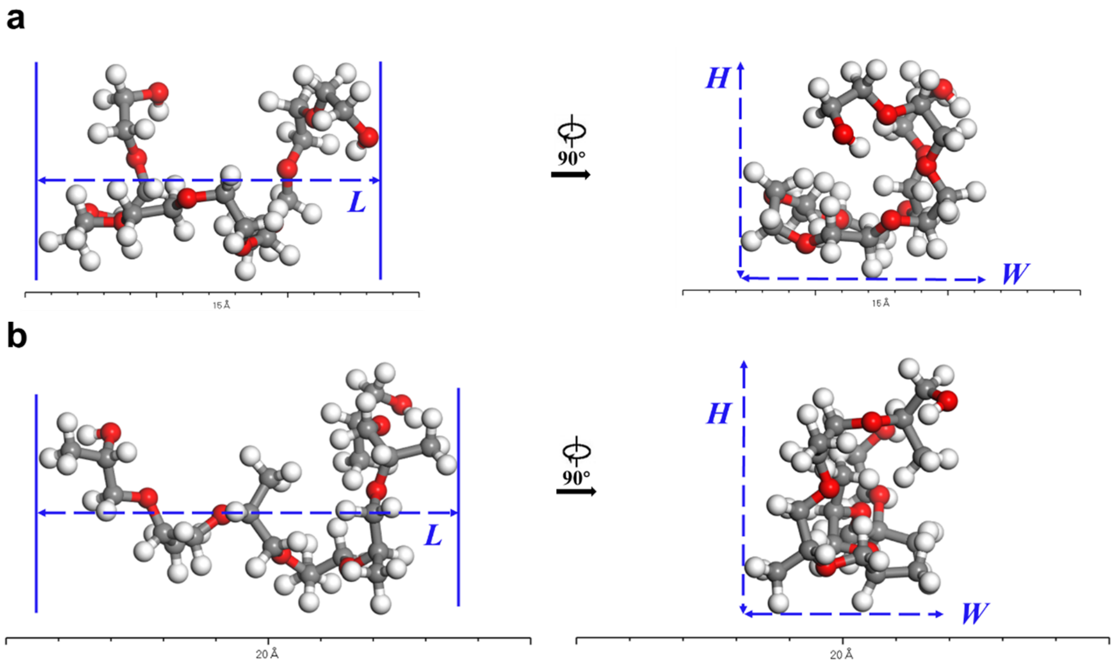

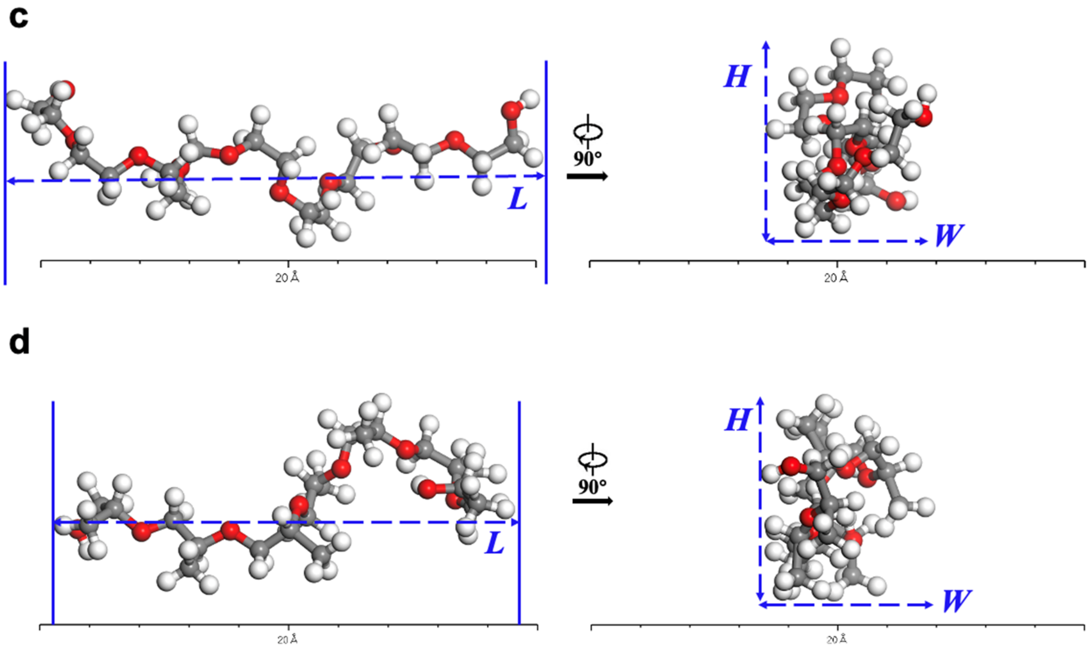

Figure S3. Dimensional properties of polymers in methanol solutions. *L*, *W*, and *H* represent the length, width and height of the polymer chain, respectively. (**a**) Twisted PEG molecule in methanol solution; (**b**) Twisted PPG molecule in methanol solution. (**c**) Stretched PEG molecule in methanol solution; (**d**) Stretched PPG molecule in methanol solution.

S.2 Vapor Pressure Measurement [1]

The vapor pressure of MeOH, PEG-400 in MeOH solution and PPG-400 in MeOH solution were measured by our laboratory-developed highly sealed apparatus shown in Figure S4. The measurement was done at 23 ± 0.1 °C by immersing the whole apparatus into the temperature-controlled water bath.

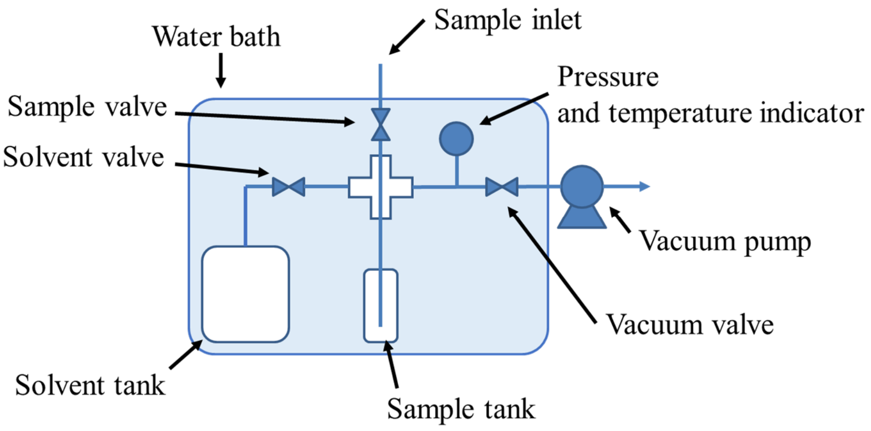

Figure S4. An apparatus for vapor pressure measurement.

S.3 Characterization of Hollow Fiber Support [2]

The mean pore size of the polyketone hollow fiber support was measured using a capillary flow porometer (CFP-1200AX; Porous Materials, Inc., USA) according to the Japanese Industrial Standards (JIS) K3832 [3]. The porosity of the polyketone HF support was measured by a gravimetric method [4]. The weights of 10 pieces of 10 cm-long HF were measured using an electronic balance. The porosity of HF, ϕ (%), was obtained by Eq. (S1), where *W* (g) was the measured weight of the HFs; *ID* (cm) and OD (cm) were the

inner and outer diameters of the HF, respectively; and ρ_{PK} the density (g/cm^3) of the polyketone, which was taken as 1.3 g/cm^3 [5].

$$\phi = 100 \times \left[1 - \frac{W}{10}\left\{\frac{4}{\rho_{PK} \times 10 \times (OD^2 - ID^2) \times \pi}\right\}\right] \quad \text{(S1)}$$

Table S1. HF support characteristics [2]. Error is the probable error.

	HF support
Inner diameter (μm)	488 ± 5
Outer diameter (μm)	784 ± 3
Thickness (μm)	148 ± 3
Porosity (%)	74.3 ± 0.2
Mean pore size (nm)	110 ± 4

S.4 Characterization of the Selective Layer [2]

The selective layer on the bore surface was characterized by ATR-FTIR. Figure 4 shows the ATR-FTIR spectra of bore surface before and after interfacial polymerization. The three new peaks at 1541, 1610, 1663 cm^{-1} in the spectrum of the TFC-HF membranes can be assigned to amide II band, aromatic amide and amide I band, respectively [6]. These peaks revealed the presence of interfacially polymerized polyamide layer at the bore surface.

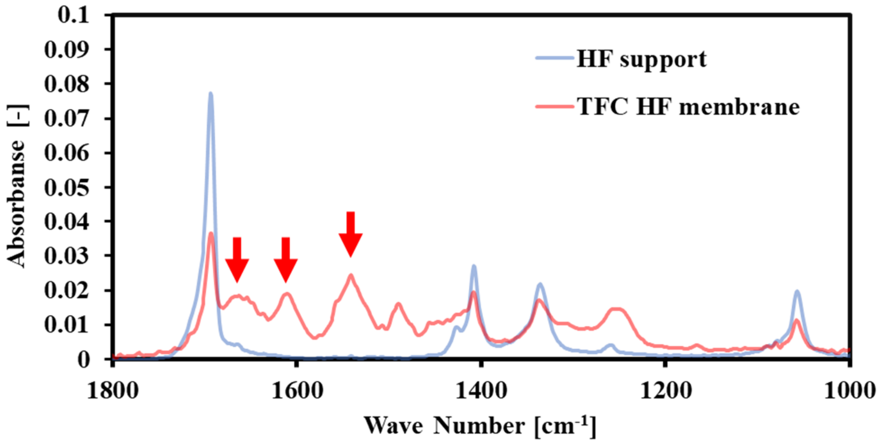

Figure S5. ATR-FTIR of the bore surface before (HF support) and after the interfacial polymerization (TFC-HF membrane) [2].

References

1. Li, J.; Gonzales, R.R.; Takagi, R.; Chen, Y.C.; Matsuoka, A.; Deng, L.; Matsuyama, H. Continuous purification of drugs by ionic liquid-drawn organic solvent forward osmosis and solute recovery. *Environ. Chem. Lett.* **2024**, *22*, 29. https://doi.org/10.1007/s10311-023-01641-y.
2. Takada, R.; Takagi, R.; Matsuyama, H. High-degree concentration organic solvent forward osmosis for pharmaceutical pre-concentration. *Membranes* **2024**, *14*, 14. https://doi.org/10.3390/membranes14010014.
3. *JIS K3832*; Testing Methods for Bubble Point of Membrane Filters. Japanese Industrial Standard: Tokyo, Japan, 1990 *JIS K3832*; Testing Methods for Bubble Point of Membrane Filters. Japanese Industrial Standard: Tokyo, Japan, 1990
4. Shibuya, M.; Yasukawa, M.; Mishima, S.; Tanaka, Y.; Takahashi, T.; Matsuyama, H. A thin-film composite-hollow fiber forward osmosis membrane with a polyketone hollow fiber membrane as a support. *Desalination* **2017**, *402*, 33. https://doi.org/10.1016/j.desal.2016.09.022.
5. Sato, D.; Kaneda, M.; Komatsu, T. Polyketone Porous Film. Patent No. WO2013/035747, 14 March 2013.

6. Tang, C.Y.; Kwon, Y.N.; Leckie, J.O. Effect of membrane chemistry and coating layer on physiochemical properties of thin film composite polyamide RO and NF membranes I. FTIR and XPS characterization of polyamide and coating layer chemistry. *Desalination* **2009**, *242*, 149. https://doi.org/10.1016/j.desal.2008.04.003.
